# Artificial Neural Network Modelling of Photodegradation in Suspension of Manganese Doped Zinc Oxide Nanoparticles under Visible-Light Irradiation

**DOI:** 10.1155/2014/726101

**Published:** 2014-11-04

**Authors:** Yadollah Abdollahi, Azmi Zakaria, Nor Asrina Sairi, Khamirul Amin Matori, Hamid Reza Fard Masoumi, Amir Reza Sadrolhosseini, Hossein Jahangirian

**Affiliations:** ^1^Material Synthesis and Characterization Laboratory, Institute of Advanced Technology, Universiti Putra Malaysia, 43400 Serdang, Selangor, Malaysia; ^2^Chemistry Department, Faculty of Science, University of Malaya, 50603 Kuala Lumpur, Malaysia; ^3^Department of Chemistry, Faculty of Science, University Putra Malaysia, 43400 Serdang, Selangor, Malaysia; ^4^Wireless and Photonics Networks Research Center (WiPNET), Faculty of Engineering, Universiti Putra Malaysia, Serdang, Malaysia; ^5^Department of Chemical and Environmental Engineering, Faculty of Engineering, Universiti Putra Malaysia (UPM), 43400 Serdang, Selangor, Malaysia

## Abstract

The artificial neural network (ANN) modeling of *m*-cresol photodegradation was carried out for determination of the optimum and importance values of the effective variables to achieve the maximum efficiency. The photodegradation was carried out in the suspension of synthesized manganese doped ZnO nanoparticles under visible-light irradiation. The input considered effective variables of the photodegradation were irradiation time, pH, photocatalyst amount, and concentration of *m*-cresol while the efficiency was the only response as output. The performed experiments were designed into three data sets such as training, testing, and validation that were randomly splitted by the software's option. To obtain the optimum topologies, ANN was trained by quick propagation (QP), Incremental Back Propagation (IBP), Batch Back Propagation (BBP), and Levenberg-Marquardt (LM) algorithms for testing data set. The topologies were determined by the indicator of minimized root mean squared error (RMSE) for each algorithm. According to the indicator, the QP-4-8-1, IBP-4-15-1, BBP-4-6-1, and LM-4-10-1 were selected as the optimized topologies. Among the topologies, QP-4-8-1 has presented the minimum RMSE and absolute average deviation as well as maximum R-squared. Therefore, QP-4-8-1 was selected as final model for validation test and navigation of the process. The model was used for determination of the optimum values of the effective variables by a few three-dimensional plots. The optimum points of the variables were confirmed by further validated experiments. Moreover, the model predicted the relative importance of the variables which showed none of them was neglectable in this work.

## 1. Introduction

According to the last report of united nation world water development, most untreated industrial wastewater which contains several kinds of organic pollutants such as phenolic compounds is flowing into the productive lands, surface, and underground water sources [1]. To prevent the hazardous materials from entering into the environment, the effective and environmental compatibility removal methods are attracting the attentions. Therefore, several chemical, physical, and biological methods have been applied to remove the pollutants by using chemical coagulation, oxidation, flocculation, precipitation, froth floatation, reverse osmosis, and biological techniques [2, 3]. However, the chemical methods are unable to mineralize all the organics and also generate new environmental pollutants [4]. In the same trend, the biological methods are slow, selective, pH, and temperature sensitive [5, 6]. The physical methods such as adsorption techniques are unable to remove the hazardous from the environment. On the other side, advanced oxidation processes (AOPs) such as heterogeneous photocatalytic processes including photocatalyst, Fenton, photo-Fenton, and electrooxidation are powerful and nonselective methods that have been used to convert the persistent organic pollutants to an environmental friendly product [7–9]. Among the various AOPs methods, the heterogeneous photocatalytic process has been succeed due to its ability for destroying a wide range of the pollutants at ambient temperature and pressure without generation of harmful intermediates [10–17]. The processes use a catalyst that is active under UV or visible-light irradiation to generate hydroxyl radical [18]. Zinc oxide (ZnO) is well-known nontoxic semiconductor materials that has been used as heterogeneous photocatalyst to investigate water purification [19, 20]. ZnO has facilitated several degradations of the water organic contaminants under UV irradiation [21, 22]. In addition, the grate advantages of ZnO are absorption of a large fraction of the solar spectrum (sunlight) which is free and available around the world [23]. Sunlight consists of 47% visible-light with wavelength of 400 to 700 nm or energy of 1.77 to 2.76 eV. Thus, visible-light could be an excellent source of energy for the photocatalytic activity. However, the photo activity of ZnO under the energy was very low due to its high direct band gap energy, 3.2 eV [19, 20]. Therefore, several methods of synthesis have been examined to improve the band gap such as transition metals doped ZnO [24–29]. As the absorption spectra red shift has showed, the 3D orbital of the metals goes between valence band (VB) and conduction band (CB) of the semiconductors in doping process [30, 31]. It depends on the energy of the 3D orbitals, they overlap with the VB or CB of the semiconductors [32]. For the reason, the electrons are excited from VB (the 3D overlapped VB) to CB (3D overlapped CB) during irradiation process. On the other hand, the energy of manganese (Mn) 3D orbitals is very close to the VB of ZnO which easily overlap to decrease the *E*_*g*_ [10, 17]. In our previous work, Mn doped ZnO was synthesized and applied for degradation of organic pollutants [29]. The photodegradation was studied by one variable at a time technique with the effective variables of irradiation times, pH, photocatalyst amount, and concentration of the pollutants [33, 34]. The problem is that the technique varies one of the parameters while the other terms are kept constant during the multivariate performance. Therefore, it has adverse effects on the photodegradation that should be studied by multivariate methods [17, 21]. Moreover, the kinetic determination of the process is quite complicated by consideration of the mass transfer, the radiant energy balance, the spatial distribution of the absorbed radiation, and mechanisms of the photochemical degradation [35]. The photodegradation as a process consists of input factors such as effective variables and the efficiency as output response. The changing amount of the effective variables affects the value of the efficiency. Therefore, the amount of the variable could be optimized to achieve the maximum efficiency that is free of the mentioned complexities. The known multivariate methods that used to optimize the productive process included response surface methodology (RSM) and artificial neural network (ANN) [35, 36]. The RSM designs the related experiments and then fits the observed results of performed design to appropriate polynomial and suggests the qualified model for more validation. The model as a mathematic equation indicates the relationships between variables themselves, variables and response(s). Thereafter, the validated model is used to optimize the effective variables to achieve the maximum yield of the products. However, the method is involved with the complicated statistical calculation such as analysis of variance, fitting, and regression process for modeling process [37, 38]. On the other hand, ANNs have been widely used for modeling of chemical and biochemical reaction process [39–43]. The ANN modeling has been reliable, robust, and salient characteristics in capturing the nonlinear relationship between the input and output variables which is free of complexities. In this work, the multilayer feed-forward neural network was used to model* m*-cresol photodegradation in manganese doped ZnO nanoparticles (Mn doped ZnO NPs) suspension under visible-light irradiation. The cresol is widely used in several manufacturing products with high water solubility which has been listed as priority pollutants, persistent toxic chemical, and a significant threat to the environment [44, 45]. The input effective operational parameters were including irradiation time, pH, photocatalyst amount, and concentration of the cresol while the efficiency % was the only response as output.

## 2. Materials and Methods

The chemicals of this work were obtained from Merck and were used without further purification. The* m*-cresol (99%) was used as organic water pollutant while H_2_SO_4_ (95%–97%) and NaOH (99%) were applied to set the appropriate pH. The Mn doped ZnO NPs with average particles size 35 nm, bang gap energy, 2.2 eV, and surface area 35 m^2^g^−1^ were used as photocatalyst. The photocatalyst was synthesized by precipitation method in absolute alcohol according to our published work [29]. To degrade the pollutant, the various concentrations of* m*-cresol were mixed with appropriate amount of the photocatalyst in 500 mL deionized water. The mixture (suspension) solution was irradiated by a Philips lamp (23 watts) as light source in a batch homemade photoreactor that was used in our previous work [46]. The suspension was magnetically stirred during the irradiation at 200 rpm. Moreover, air was blown into the solution by using an air pump at a flow rate of 150 mL/min to increase solution fluidization, access oxygen, volatile the produced gas (CO_2_), and keep the temperature at around 25°C. During the performance, samples were withdrawn from the bulk solution at specific time intervals and centrifuged at 14000 rpm for 20 min and then they filtered through 0.2 *μ*m PTFE filter to measure the remained concentration of the* m*-cresol. The measurement was carried out by a Shimadzu UV-1650 PC and a TOC-VCSN analyzer, respectively. In addition, the initial catalyst absorption and photocatalyst were investigated in dark and absence of the photocatalyst at normal pH (7.5) that were considered in the efficiencies calculation [47]. The efficiencies were used as actual responses for modeling of the photodegradation which was carried out by Neural Power Software version 2.5 [48, 49]. The total of 31 experiment points have been randomly splitted into training (15 points), testing (8 points) and validation (6 points) data sets (Table 1) by using the facilitated option in the software. The training was used to compute and ensure robustness of the network parameters while the testing stage was used as control errors to avoid overfitting [50]. The validation data which was excluded from training and testing considered to assess the predictive ability of the generated model [51].

## 3. Theory of the Work

### 3.1. The Theory of ANN

ANNs are semiempirical multivariate methods that are used in mathematic free fictionalization of the complicated productive process. The networks contain input, hidden, and output layers which are made of several nodes. The nodes are connected by multilayer normal feed-forward or feed-back connection formula [52]. The nodes are simple artificial neurons which stimulates the behavior of biological neural networks. The hidden layer could be more than one parallel layer however the single hidden layer is universally suggested. In the network, the nodes of particular layer are connected to the nodes of the next layer from left to right by feed-forward formula. The nodes of input layer are qualified by sending data via the special weights to the nodes of hidden layer and then to the output layer [52, 53]. The qualification is carried out by associated weights during learning process by well-known mathematic algorithms.

### 3.2. The Learning Process

The learning process determines the number of nodes in the hidden layer (topology) by using trial and error calculation. The calculation is examined from one to “*n*” nodes to discover the architecture with minimum root mean square error (RMSE) by using testing data set and particular algorithm. In learning process, the input layer acts as distributor for the hidden layer and the inputs and output of the hidden layer are multiplied by weighted summation as follows:

(1) $$\begin{array}{c} S=\overset{nh}{\underset{i=1}{\sum }}\left(b-W_{i}I_{i}\right), \end{array}$$

where *S* is summation, *b* is a bias [54], *I*_*i*_ is the *i*th input to hidden neuron, and *W*_*i*_ is the weight associated with *I*_*i*_. The bias shifts the space of the nonlinearity properties [55]. Therefore, the outputs of the hidden layer act as inputs to final layer (output) which are undergoing a transfer function. The popular transfer function is the logarithmic sigmoid for both hidden and output layers that is bounded from 0 to 1 [56]. The sigmoid bounded area is used to normalize the input and output data that is provided by the software scaling. The scaled data are passed into the first layer and propagated to hidden layer and finally meet the output layer of the network by iterative procedure. The iteration is an act of repeating a process to approach a desire result. The results of iteration are used as starting point of next iteration. For example, when the results of last iteration become almost equal to the results of previous iteration, the process will be terminated. The iteration process is continued by self-similarity method as follows [57]:

(2) $$\begin{array}{c} S\left(B\right)=\overset{m}{\underset{I=1}{\sum }}\left[y_{i}-f{\left(x_{i}\beta \right)}^{2}\right], \end{array}$$

where “*m*” is an empirical data pairs of independent and dependent variables such as (*x*_*i*_, *x*_*i*_) and *f*(*x*_*i*_, *β*) is the model curve. In self-similarity process, the *β* parameter of *f*(*x*_*i*_, *β*) is optimized by minimizing the sum of the squares. As a result, the main aim of the learning process is to find the weights for minimizing the error of (RMSE) which is obtained from difference between network prediction and actual responses as follows:

(3) $$\begin{array}{c} \text{RMSE}={\left(\frac{1}{n}\overset{n}{\underset{i=1}{\sum }}{\left(y_{i}-y_{di}\right)}^{2}\right)}^{1/2}, \end{array}$$

where “*n*” is number of the points, *y*_*i*_ is the predicted values, and *y*_*di*_ is the actual values. To avoid random correlation due to the random initialization of the weights, the examination of each node is repeated several times. Among the repeated examination, the architecture with lowest RMSE is selected for each node. The RMSE of the architectures are compared to find the best topology for the particular algorithm. The topology is architecture with minimum relative RMSE. For more certainty, the *R*-square (*R* ^2^) (see (4)) and absolute average deviation (AAD) (see (5)) are calculated by performance of the topology for training and testing data sets:

(4) $$\begin{array}{c} R^{2}=1-\frac{\sum_{i=1}^{n}{\left(y_{i}-y_{di}\right)}^{2}}{\sum_{i=1}^{n}{\left(y_{di}-y_{m}\right)}^{2}} \end{array}$$

(5) $$\begin{array}{c} \text{AAD}=\frac{1}{n}\overset{n}{\underset{I=1}{\sum }}\frac{\left|y_{i}-y_{di}\right|}{y_{di}}, \end{array}$$

where “*n*” is the number of points, *y*_*i*_ is the predicted value, *y*_*di*_ is the actual value, and *y*_*m*_ is the average of the actual values. The learning process is carried out for different algorithms to obtain the best topology. Then the RMSE, AAD, and *R* ^2^ of the topologies are compared to find the optimized topology that is selected as provisional model for the process. The model is evaluated by validation data set (Table 1). Thereafter, it is used for navigation of the process that determines the optimum and importance of the input variables to maximize the yield of the process.

## 4. Results and Discussion

### 4.1. The Modeling Process

The network of the photodegradation process contains input, hidden, and output layers which they are made of one or more number of nodes. The structure of the input and output layer was determined by number of the effective variables and the efficiency of the photodegradation while the structure of the hidden layer was determined by the modeling. The effective variables included temperature, pH, photocatalyst amount, and concentration of* m*-cresol.

#### 4.1.1. The Structure of the Hidden Layer

**Figure 1:**
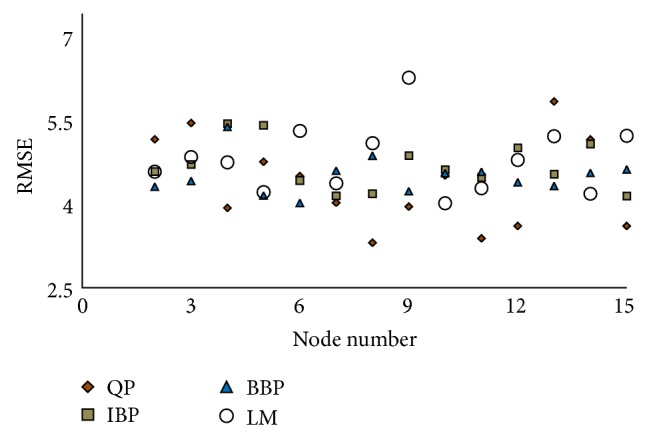
The selected RMSE versus node number of the photodegradation network's hidden layer for QP, IBP, BBP, and LM algorithm. The smallest RMSE belong to node of 8 (QP), 15 (IBP), 6 (BBP) and 10 (LM).

To obtain the structure of the hidden layer, 15 architectures that contained 1 to 15 nodes were examined for quick propagation (QP), Incremental Back Propagation (IBP), Batch Back Propagation (BBP), and Levenberg-Marquardt (LM) algorithms. The examination was repeated 10 times for each node by testing data set. Then, among the 10 repetitions, the architecture with the smallest RMSE was selected for each node. Therefore, 15 architectures were considered for each algorithm illustrated in Figure 1 to find the optimized topologies. Figure 1 plots the value of RMSE versus the node number for QP, IBP, BBP, and LM algorithms. For each algorithm, one of the 15 architectures has presented minimum RMSE (topology) considered for more evaluation. The hidden layer's node numbers of selected topologies were 8, 15, 6, and 10 for QP, IBP, BBP, and LM logarithms, respectively. The evaluation of the topologies was carried out by comparison of minimum RMSE and AAD as well as maximum *R* ^2^ to discover the provisional model of the photodegradation. The comparison of the RMSE proved that the QP with 4 nodes in input, 8 nodes in hidden, and 1 node in output layer (QP-4-8-1) has presented the minimum root mean squared error (Figure 1).

**Figure 2:**
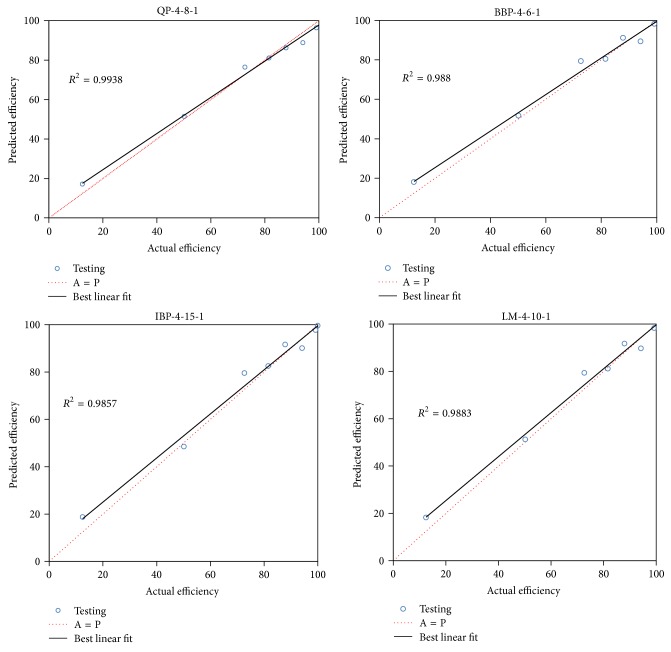
The scatter plots of the predicted efficiency versus actual efficiency for testing data set that shows the performed *R* ^2^ of optimized topologies, QP-4-8-1, BBP-4-6-1, IBP-4-15-1, and LM-4-10-1.

Then, the performed results of the topologies were used to calculate *R* ^2^ (see (4)) and ADD (see (5)). To calculate the *R* ^2^, the prediction of the topologies and actual values of the efficiency were plotted for testing data set in Figure 2. As the scatter plots showed, the topology of QP-4-8-1 has presented the highest *R* ^2^, 0.993, that, in comparison with other topologies, has the best performance.

**Figure 3:**
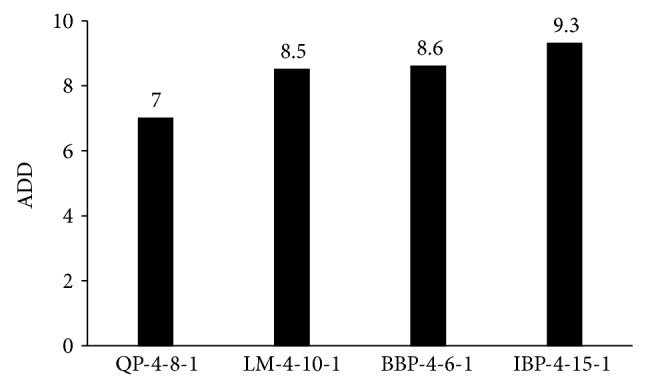
The AAD of QP-4-8-1, BBP-4-6-1, IBP-4-15-1, and LM-4-10-1 topologies in testing data set.

Moreover, the AAD of the topologies in testing data set was calculated for QP-4-8-1, IBP-4-15-1, BBP-4-6-1, and LM-4-10-1 (Figure 3). As shown, the lowest value of the AAD has also belonged to topology of QP-4-8-1. As a result, the topology of QP-4-8-1 was pioneer in minimum RMSE and AAD as well as at maximum of *R* ^2^ among those topologies for testing data sets. Therefore, QP-4-8-1 was selected as final optimum provisional model of the photodegradation for validation test.

#### 4.1.2. Validation of the Selected Model

**Figure 4:**
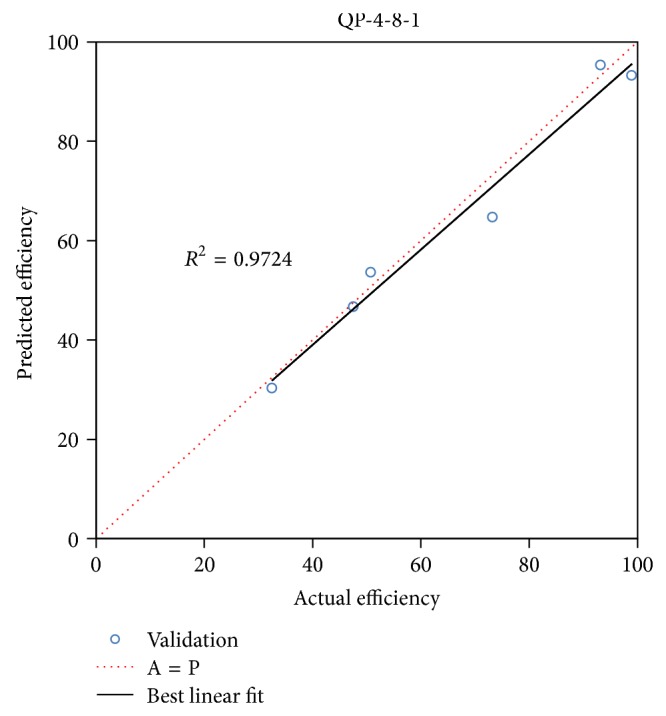
The validation test of the provisional model (QP-4-8-1) which consists of actual and predicted photodegradation efficiency as well as the best linear fit and *R*-square.

The provisional model (QP-4-8-1) was validated by 6 experimental points excluded from training and testing data sets (Table 1). The validation was investigated by scatter plots of the model prediction versus actual values of the photodegradation efficiency (Figure 4). As demonstrated, the *R* ^2^, 0.972, of the performance was quite close to 1 that confirmed the model is significant. In addition, the RMSE and AAD of the performed validation included 4.574 and 5.668, respectively, which proved the great predictive accuracy of the model.

#### 4.1.3. The Final Model of the Photodegradation

**Figure 5:**
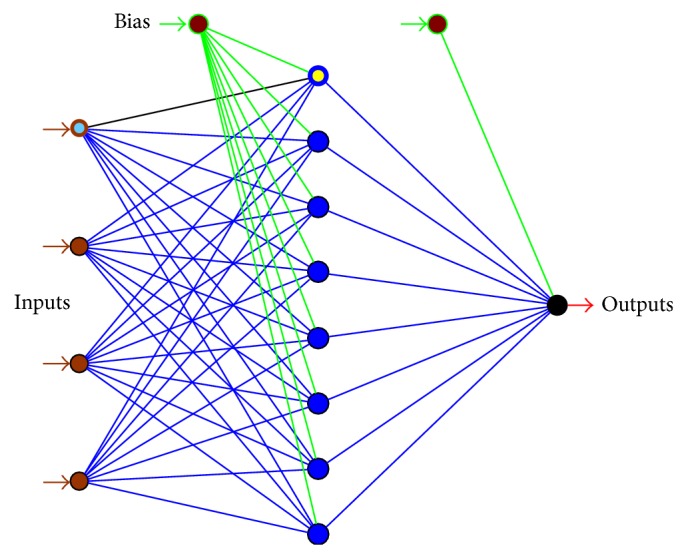
The structure of the photodegradation final model of QP-4-8-1 that consists of 4 nodes in input layer, 8 nodes in hidden layer, and 1 node in output layer as response.

Figure 5 shows the structure of QP-4-8-1 topology as final model for* m*-cresol photodegradation. The model consists of three layers that included input, hidden, and output layers. The input layer with 4 nodes of effective variables acts as distributor for the hidden layer with 8 nodes. The inputs as well as output of hidden nodes are multiplied by the appropriate weights [55]. Then the nodes outputs of hidden layer are transferred to output layer by using log-sigmoid transfer function (see (6)) [58]. The function normalizes transfers the data between the layers [56]:

(6) $$\begin{array}{c} f\left(x\right)=\frac{1}{1+\exp\left(-x\right)}, \end{array}$$

where *f*(*x*) is the hidden output neurons. Therefore, QP-4-8-1 was used to navigate the process for determination of optimum and importance values of the photodegradation input variables.

### 4.2. The Model Applications of the Photodegradation

As a short overview, the modeling process optimized the topologies of different learning algorithms by using testing and training data sets. Then the best topology with optimum *R* ^2^, RMSE, and AAD was selected as provisional model for more evaluation. The adequacy of the selected model (QP-4-8-1) was evaluated by validation data set. The validation model QP-4-8-1 was used to navigate the photodegradation. The navigation has contained graphical optimization and the relative importance of the input effective.

#### 4.2.1. The Variables Graphical Optimization

**Figure 6:**
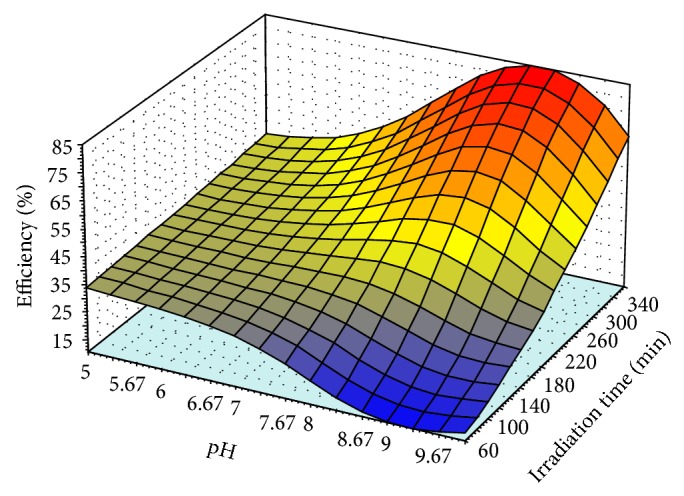
The variation of the photodegradation efficiency in pH 5 to pH 10 and irradiation time (60 to 360 min), the amount of photocatalyst (2 g/L) and* m*-cresol (45.5 mg/L) were at middle of their levels.

**Figure 7:**
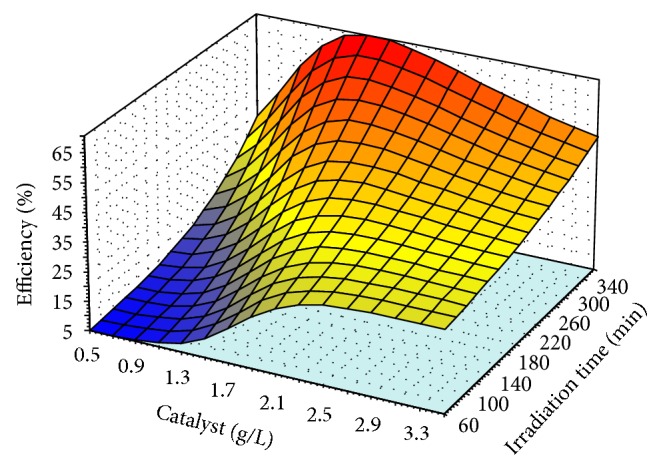
The efficiency variation of the photodegradation with varying photocatalyst from 0.5 to 3.5 g/L and irradiation time (60−360 min); the pH was 7.5 and* m*-cresol was 45.5 mg/L.

**Figure 8:**
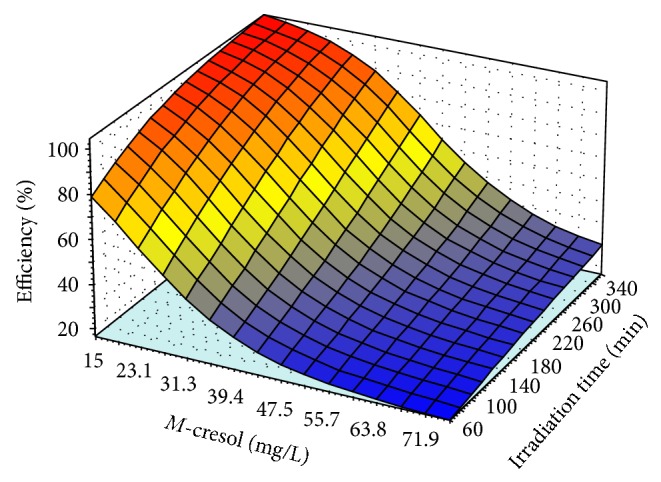
The variation of the efficiency in different concentration of* m*-cresol from 25 to 80 mg/L with irradiation time (60−360 min); the pH was 7.5 and amount of the photocatalyst was 2 g/L.

The validated model, QP-4-8-1, simulated the behavior of the photodegradation without further requirement of mathematic knowledge (Figures 6 to 8). The simulations consist of effect of nonlinear relationship of two variables on photodegradation efficiency which is graphically presented by three dimensional plots (3D) while the other parameters were kept constant at the middle of their levels' values. The values of irradiation time were 210 min, pH was 7.5, photocatalyst amount was 2 (g/L), and concentration of* m*-cresol was 45.5 (mg/L).

Figure 6 shows the variation of the efficiency in pH 5 to pH 10 during irradiation time 60 to 360 min while the amount of photocatalyst and* m*-cresol were kept constant at 2 g/L and 45.5 mg/L, respectively. As shown, the efficiency was increased up to pH 9 and thereafter it was decreased for irradiation time 300 to 360 min. Therefore, the maximum surface of the 3D plot has been demonstrated at pH (8 to 9) and the 340 to 360 min of irradiation time.

Figure 7 shows the photodegradation efficiency versus the photocatalyst amount from 0.5 to 3.5 g/L and irradiation time (60−360 min). The pH and* m*-cresol were kept constant 7.5 and 45.5 mg/L, respectively. As shown, the efficiency was increased up to 1.3 g/L of the photocatalyst, then it was constant up to 1.7 g/L, and finally it was decreased. Therefore, the optimum value of the photocatalyst was in the level of 1.3 to 1.7 g/L.

Figure 8 shows the efficiency of the photodegradation in different concentration of* m*-cresol (25 to 80 mg/L) in level of irradiation time from 60 to 360 min while the pH, 7.5, and the photocatalyst, 2 g/L, were kept constant. As the 3D plot demonstrates, the efficiency was continually decreasing with increasing* m*-cresol concentration. However, the removed* m*-cresol was more than 80% of 45.5 mg/L at the end of the irradiation time (340–360 min). Therefore, the maximum amount of removed* m*-cresol was 42 mg/L.

The optimum value of the irradiation time was investigated by Figures 6 to 8 obtained in levels 340 to 360 min at the middle values of the other variables levels such as the photocatalyst, pH, and* m*-cresol concentration. As a result of Figures 6 to 8, the optimum levels of the effective variables were photocatalyst (1.3–1.7 g/L), pH (8-9),* m*-cresol (40–45 mg/L), and irradiation time (340–360 min). To predict the optimum points of these levels, the desirable condition such as maximum* m*-cresol's concentration, minimum amount of the photocatalyst, and pH value at the end of the irradiation time was considered as input for the model. The model prediction included 1.3 g/L photocatalyst, 42 mg/L* m*-cresol, pH 8, and 360 min of the irradiation time while the predicted efficiency was 100%. Then this condition was evaluated by further experiments and observed efficiency was 99% (almost all* m*-cresol was removed) that confirmed the improvement in comparison with other previous works [59].

#### 4.2.2. Importance of the Effective Variables

**Figure 9:**
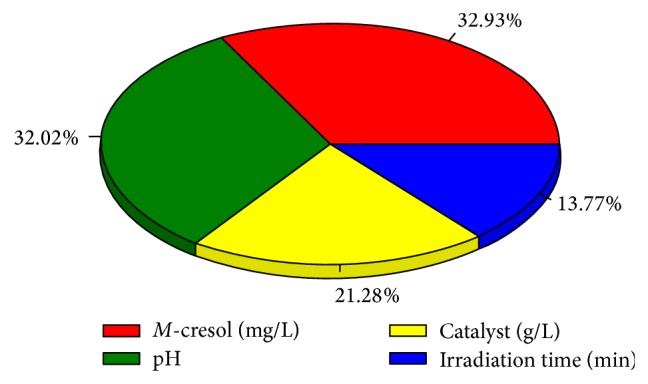
The relative importance of the photodegradation input variables such as irradiation time, pH amount of the photocatalyst, and* m*-cresol's concentration.

The relative importance of the effective variables in the optimum levels was determined by the model as presented in Figure 9. As demonstrated, the greatest importance belonged to* m*-cresol (32.93%) and pH (32.02%). However, the effects of other variables such as photocatalyst (21.28%) and irradiation time (13.77%) were also important for the efficiency. As a result, the selected variables were effectiveness and none of them was neglectable in this photodegradation.

#### 4.2.3. The Model Multivariate Navigation

To navigate the photodegradation, the model (Figure 5) was used to determine the optimum levels, predict optimum points, and obtain the importance of the effective variables. The variables were initially used in a wide range and identical importance and without any considered points. The obtained information is presented by Table 2. The optimum levels were achieved by 3D plots in graphical vision (Figures 6 to 8). The optimum points of the variables in the optimized narrow levels were firstly predicted by the model and verified by further experiment with 1% error. Moreover, the model determined the relative importance of the variables which showed none of them were neglectable in this work.

## 5. Conclusions

The AAN modeling of* m*-cresol photodegradation was carried out to determination of optimum and importance values of the effective variables to achieve maximum efficiency. The photodegradation was performed in synthesis Mn doped ZnO suspension and under visible-light irradiation. The input considered effective variables of the photodegradation were irradiation, time, pH, photocatalyst amount, and concentration of* m*-cresol while the efficiency was the only response as output. The performed experiments were designed in three data sets such as training, testing, and validation that were randomly splitted by the software's option. To obtain the optimum topologies, AAN was trained by QP, IBP, BBP, and LM algorithms for testing data set. The topologies were determined by the indicator of minimized RMSE for each algorithm. According to the indicator, the QP-4-8-1, IBP-4-15-1, BBP-4-6-1, and LM-4-10-1 were selected as the optimized topologies. Among the topologies, QP-4-8-1 has presented the minimum RMSE and AAD as well as maximum *R* ^2^. Therefore, QP-4-8-1 was selected as final model for the photodegradation navigation. The model was used for determination of the optimum values of the effective variables to achieve the maximum efficiency by using graphical vision. The predicted optimum points of the variables were confirmed by further validated experiments. Moreover, the model predicted the relative importance of the variables which showed none of them was neglectable in this work.

## Figures and Tables

**Table 1:** The training, testing, and validation data sets of the effective variables and actual and models predication efficiency of *m*-cresol photodegradation by Mn doped ZnO NPs.

Run	Irradiation time	pH	Photocatalyst	*m*-cresol	Efficiency	
					Actual	Predicted
Training data set						
1	360	7.63	1.5	65	38.9	37.057
2	120	7.63	1.5	35	30.86	30.435
3	360	4	1.5	35	46.4	46.457
4	360	10	1.5	35	48.7	48.922
5	360	7.63	3	35	83.7	84.001
6	360	7.63	1.7	35	95	95.864
7	240	7.63	1.5	35	65.08	66.073
8	360	8.5	1.5	35	89.3	90.323
9	360	6	1.5	35	67.2	68.333
10	360	8	1.5	35	93.4	94.741
11	360	7.63	1	35	79.9	81.33
12	300	7.63	1.5	35	81.42	82.96
13	360	7.63	1.5	55	46.80	49.625
14	360	7.63	3.5	35	72.4	80.016
15	360	7.63	1.5	15	100	109.16
Testing data set						
17	360	9	1.5	35	72.7	79.504
18	360	7.63	0.5	35	50.2	64.026
19	360	7.63	1.5	41	81.6	74.845
20	360	7.63	1.3	35	94.2	88.926
21	360	7.63	1.5	30	99.2	97.703
22	360	7.63	1.5	25	100	99.572
23	360	7.63	2.5	35	87.9	90.131
24	60	7.63	1.5	35	12.48	17.993
Validation data set						
26	360	7.63	1.5	45	73.3	62.789
27	360	7.63	1.5	35	98.98	91.939
28	360	7.63	1.5	76	32.5	28.915
29	180	7.63	1.5	35	47.55	46.747
30	360	5	1.5	35	50.8	52.441
31	360	7.63	2	35	93.2	94.923

**Table 2:** The results of multivariate modeling and optimization of *m*-cresol photodegradation under visible-light irradiation.

Variable	*m*-cresol (mg/L)	pH	Photocatalyst (g/L)	Irradiation time (min)	Efficiency (%)
Optimum level	40 to 45	8 to 9	1.3 to 1.7	340 to 460	—
Predicted point	42	8	1.3	360	100
Verified point	42	8	1.3	360	99
Importance (%)	32.93	32.02	21.28	13.77	—
